# Mild hyperthermia influence on Herceptin^®^ properties

**DOI:** 10.2478/raon-2014-0045

**Published:** 2015-03-03

**Authors:** Jean-Michel Escoffre, Roel Deckers, Noboru Sasaki, Clemens Bos, Chrit Moonen

**Affiliations:** Imaging Division, UMC Utrecht, Utrecht, the Netherlands

**Keywords:** mild hyperthermia, anticancer antibody, Herceptin^®^, breast cancer

## Abstract

**Background:**

Mild hyperthermia (mHT) increases the tumor perfusion and vascular permeability, and reduces the interstitial fluid pressure, resulting in better intra-tumoral bioavailability of low molecular weight drugs. This approach is potentially also attractive for delivery of therapeutic macromolecules, such as antibodies. Here, we investigated the effects of mHT on the stability, immunological and pharmacological properties of Herceptin^®^, a clinically approved antibody, targeting the human epidermal growth factor receptor 2 (HER-2) overexpressed in breast cancer.

**Results:**

Herceptin^®^ was heated to 37°C (control) and 42°C (mHT) for 1 hour. Formation of Herceptin^®^ aggregates was measured using Nile Red assay. mHT did not result in additional Herceptin^®^ aggregates compared to 37°C, showing the Herceptin^®^ stability is unchanged. Immunological and pharmacological properties of Herceptin^®^ were evaluated following mHT using HER-2 positive breast cancer cells (BT-474). Exposure of Herceptin^®^ to mHT preserved recognition and binding affinity of Herceptin^®^ to HER-2. Western-blot and cell proliferation assays on BT-474 cells showed that mHT left the inhibitory activities of Herceptin^®^ unchanged.

**Conclusions:**

The stability, and the immunological and pharmacological properties of Herceptin^®^ are not negatively affected by mHT. Further *in-vivo* studies are required to evaluate the influence of mHT on intra-tumoral bioavailability and therapeutic effectiveness of Herceptin^®^.

## Introduction

Breast cancer is the first most common cancer (464,000 cases in 2012) and is the third cause of death from cancer for women (131,000 in 2012) in Europe.^1^ In 25% to 30% of breast cancer, human epidermal growth factor receptor 2 (HER-2) is amplified and overexpressed, which is associated with poor prognostic factors as shown by its highly proliferative and often high-grade histology.^2^,^3^ Moreover, women with HER-2 positive breast cancer are more likely to develop visceral and central nervous system metastases than those with HER-2 negative breast cancer.^4^ Activation of HER-2 receptors triggers several signaling pathways including phosphoinositide 3-kinase (PI3K) and mitogen-activated protein kinase (MAPK) cascades which are intricately involved in multiple processes of breast cancer pathogenesis.^5^

Over the past decades, advances in pharmaceutical research have led to the development of therapeutic monoclonal antibodies (mAbs) which are able to discriminate between healthy and malignant tissue, unlike most conventional cancer treatments.^6^ FDA and EMEA have approved many mAbs for treating hematological malignancies (*e.g.,* Zevalin^®^, Rituxan^®^) and solid tumors (*e.g.,* Erbitux^®^, Vectibix^®^) for clinical use. Among these antibodies, Herceptin^®^ is a clinically approved mAb for treating HER-2 positive breast cancer that specifically binds to the juxtamembrane portion of the extracellular domain of HER-2 receptors. Herceptin^®^ exerts its antitumor effect by two main mechanisms: (i) The Herceptin^®^ binding to the HER-2 receptors induces the internalization and the degradation of HER-2 receptors, therefore inhibiting the phosphorylation of HER-2 receptors.^7^,^8^ Thus, the PI3K and MAPK signaling pathways are deactivated, promoting cell cycle arrest and apoptosis. (ii) Herceptin^®^ triggers an ADCC (antibody-dependent cellular cytotoxicity) immune response.^9^,^10^ Furthermore, Herceptin^®^ may suppress tumor neo-angiogenesis by modulating the effects of pro- and anti-angiogenic factors.^11^ In clinical trials, Herceptin^®^ has shown to decrease mortality in patients with early HER-2 positive and metastatic breast cancer, either as a monotherapy^12^ or combined with chemotherapy^13^–^15^ or with radiotherapy.^16^,^17^

Nevertheless, Herceptin^®^-based immunotherapy, as well as other mAb anticancer treatments, is rarely curative in solid tumors. This is most probably related to their poor intratumoral (i.t.) bioavailability that is compensated for by administration of high or frequent mAb doses to achieve therapeutic effectiveness. These therapeutic doses are given systemically and are associated with undesirable side effects (*e.g.,* cardiotoxicity).^18^ The physicochemical properties of mAbs, as well as the physiological barriers of tumors, limit the success of this approach for treating solid tumors.^19^ The large size of mAbs (≈ 150 kDa) does provide a convenient and long circulatory half-life (t_1/2 _> 21 days in humans for Herceptin^®^), but on the other hand restricts its extravasation from the tumor microvasculature, as well as its penetration into tumor interstitium.^19^–^22^ To overcome these limitations, the development of efficient delivery methods is required to increase the local concentration of Herceptin^®^ at the desired site while minimizing side effects to healthy tissues.

Mild hyperthermia (41°C – 43°C for 30–60 min) shows great promise in improving the therapeutic effectiveness of drugs by acting on tumor hemodynamics: (i) increasing blood flow, thus increasing drug bioavailability in tumors^23^–^25^; (ii) increasing vascular permeability and reducing interstitial fluid pressure, resulting in better drug penetration.^12^,^15^,^26^–^29^ Nowadays, mild hyperthermia (mHT) remains modestly explored as method for improving the i.t. bioavailability of full intact mAbs, such as Herceptin^®^. Additive or synergetic effects of mHT on mAbs delivery have been successfully reported for mAb fragments^28^ and reactive haptens with bi-functional mAb fragments.^30^ In addition, mHT enhanced antigen (Ag) expression, thus enhancing specific binding and retention of mAbs in tumors.^31^,^32^ Therefore, mHT may not only improve the i.t. bioavailability of intact mAbs, such as Herceptin^®^, but also increases the number of binding sites.

However, hyperthermia is known to induce protein aggregation. Indeed, therapeutic proteins, such as mAbs, are physico-chemically unstable and aggregation is one of the key features compromising their physical stability.^33^ As previously reported, protein aggregation induces a reduction or loss in biological activity, leading to a decrease in their therapeutic potential.^34^ Because the Herceptin^®^ needs to be fully active when delivered to patients to be effective, the objective of the present study is to evaluate the influence of mHT on the activation of Herceptin^®^. Herein, we report on a study of the influence of mHT on the stability, and the immunological and pharmacological properties of Herceptin^®^. Our methodology, including molecular biology and immunological techniques, addresses the following questions: (i) Does mHT induce Herceptin^®^ aggregation, thus leading to a loss of anticancer activity? (ii) Are the recognition and the affinity of Herceptin^®^ to HER-2 receptors modified by mHT? (iii) Does mHT disturb the Herceptin^®^-mediated HER-2 degradation and dephosphorylation? and (iv) Are anti-proliferative properties of Herceptin^®^ altered by mHT?

## Materials and methods

### Chemicals

Propidium iodide and TO-PRO^®^-3 iodide were purchased from Sigma-Aldrich^®^ (St. Louis, MO) and Life Technologies™ Europe B.V. (Bleiswijk, Netherlands), respectively. Fluorescein isothiocyanate (FITC)-conjugated mouse monoclonal secondary antibody anti-human IgG1-Fc (Ab50473), rabbit polyclonal anti-HER 2 (ab2428), anti-pHER-2 (ab47263), anti-ACTIN (ab1801) antibodies and horseradish peroxidase (HRP)-conjugated goat polyclonal secondary anti-Rabbit IgG Fc (ab97200) were obtained from Abcam^®^ (Cambridge, UK). FITC-conjugated human IgG1 isotype control (ANC-295-040) and human IgG1 isotype control (ANC-295-010) were purchased from Adipogen^®^ International (Liestal, Switzerland).

### Cell culture

BT-474, HER-2 positive breast cancer cells were derived from a human ductal carcinoma (ATCC^®^HTB-20™; LGC Standards GmbH, Wesel, Germany). The cells were grown as a monolayer in Roswell Park Memorial Institute Medium (RPMI-1640; Sigma-Aldrich^®^) supplemented with 10% heat-inactivated fetal calf serum (FCS; Sigma-Aldrich^®^), 10 μg/mL human insulin (Life Technologies™ Europe B.V.), 100 U/mL penicillin and 100 μg/mL streptomycin (Sigma-Aldrich). The cells were routinely sub-cultured every 5 days and incubated at 37 °C in a humidified atmosphere with 5% CO_2_.

### Herceptin^®^ & mild hyperthermia

Herceptin^®^ was purchased from F. Hoffmann-La Roche Ltd (Pensberg, Germany) and supplied at a concentration of 21 mg/mL (*i.e.,* clinical dose), as a neutral aqueous solution (phosphate-buffered saline, PBS).^35^ The stock solution of Herceptin^®^ was diluted to a concentration of 0.8 mg/mL or 250 μg/mL in PBS. The diluted solution was incubated at 37°C (*i.e.,* native Herceptin^®^) and 42°C (*i.e.,* heated Herceptin^®^) for 1 h in a heat block under gentle agitation (*i.e.,* optimal mHT condition for *in-vivo* antibody delivery^28^, ^36^).

### Antibody aggregation

As previously described, antibody aggregation was evaluated using a Nile Red assay.^33^ Nile Red binds to the hydrophobic surfaces of antibodies during aggregate formation. In such a hydrophobic environment, Nile Red exhibits a strong fluorescence whereas in aqueous medium, the fluorescence is quenched. 1 μL of Nile Red solution (100 μM) was added to 100 μL Herceptin^®^ solution (0.8 mg/mL). Subsequently, the antibody solutions were incubated at 37°C (*i.e.,* native Herceptin^®^), 42°C (*i.e.,* heated Herceptin^®^) and 90°C (*i.e.,* positive control) for 1 h in a heat block under gentle agitation.

#### Aggregate counting and size

Antibody sample aliquots were placed on Kova Glasstic slides (Hycor, Garden Grove, USA) and observed using a Keyence BZ-900 fluorescence microscope (Keyence International, Mechelen, Belgium) with a 40× Plan Fluor EL objective (0.6 NA, Ph1). Microscopic images were processed with ImageJ software version 1.42q (NIH, Bethesda, USA).^33^ Briefly, microscopic images were first converted to 8-bit grayscale images. Subsequently, the images were calibrated using the “Set Scale” command. An intensity-based threshold was applied manually to delineate the antibody aggregates. A mask was then computed the following magnitudes in the mask were collected with “Analyze Particles” command: number of aggregates, area and mean fluorescent intensity of aggregate. Data were presented as mean ± standard deviation (SD) from five independent experiments.

#### Fluorescence measurements

Herceptin^®^ sample aliquots were placed in 96-well plates and the fluorescence was measured by a FLUOstar OPTIMA (BMG Labtech GmbH, Ortenberg, Germany). The antibody aggregation was determined by measuring the intensity of Nile Red fluorescence (Excitation wavelength: 550 ± 10 nm; Emission wavelength: 615 ± 10 nm). Data were presented as mean ± SD from five independent experiments.

### Recognition of HER-2 receptors by Herceptin^®^

The recognition of HER-2 receptors by Herceptin^®^ was assessed by indirect immunofluorescence using confocal microscopy and flow cytometry.

#### Confocal fluorescence microscopy

BT-474 cells were seeded on a glass coverslip chamber (Ibidi^®^ LLC, Verona, WI) 48 hrs before the immunofluorescence staining. They were washed with ice-cold PBS with Ca^2+^/Mg^2+^ and fixed with 4% paraformaldehyde (Sigma-Aldrich^®^) in PBS for 10 min at 4°C under gentle agitation. Subsequently, cells were washed twice with cold PBS with Ca^2+^/Mg^2+^ and incubated with PBS supplemented with 2% FCS (PBS-2% FCS) for 20 min at 4°C under gentle agitation (200 rpm). After two additional washing steps with PBS-2% FCS, they were incubated in PBS-2% FCS with native and heated Herceptin^®^, or human IgG1 isotype control (10 μg/mL), for 1 h at 4°C under gentle agitation. Cells were then washed twice and incubated in the dark for 30 min at 4°C under gentle agitation, with a FITC-conjugated secondary antibody diluted in PBS-2% FCS. Subsequently, cells were washed twice and resuspended in 0.6 mL PBS-2% FCS containing TO-PRO^®^-3 (1 μM; Life Technologies™) used to localize cell nuclei. The chamber was mounted on the Zeiss LSM 510 live-cell imaging station stage equipped with a 32 PMT meta-detector (Carl Zeiss, MicroImaging GmbH, Gottingen, Germany) and Plan-Apochromat 63× Zeiss objective (1.4 NA, oil immersion). Fifteen different images per experimental condition were acquired (Zen 2008 software, Carl Zeiss) to observe FITC-labeled plasma membrane (excitation laser: 488 nm; emission filter: BP 505–530 nm) and TOPRO^®^-3 stained nuclei (excitation laser: 633 nm; emission filter: LP 650 nm). Images of cells were analyzed with the “Measure” and “Cell counter” functions of ImageJ software. The total number of HER-2 positive breast cancer cells and the associated fluorescence intensity were determined for each image (N = 400 cells/experimental condition). Data were presented as mean ± SD from three independent experiments.

#### Flow cytometry:

BT-474 cells were harvested using 2 mM EDTA in PBS, washed with cold PBS solution and incubated in PBS-2% FCS for 20 min at 4°C under gentle agitation. Subsequently, cells were centrifuged (150 g, 5 min, 4°C) and washed twice with PBS-2% FCS. The cells were then incubated in PBS-2% FCS with native or heated Herceptin^®^ (10 μg/mL) for 1 h at 4°C under gentle agitation. Human IgG1 isotype control (10 μg/mL) was used as nonbinding antibody control. After two additional washing steps, cells were incubated with FITC-conjugated anti-human IgG1-Fc antibody diluted in PBS-2% FCS in the dark, for 30 min at 4°C under gentle agitation. Cells were then washed twice and resuspended in 0.4 mL of PBS-2% FCS containing propidium iodide (0.1 μg/mL; Sigma-Aldrich^®^) to stain dead cells. Dot plots were recorded using the BD FACSCanto II flow cytometer (BD Biosciences, Breda, Netherlands) and analyzed using the BD FACS Diva software (v6.1.3., BD Biosciences) supplied by the manufacturer. A minimum of 10,000 events were analyzed to generate each dot plot. Data were presented as mean ± SD from three independent experiments.

### Affinity binding of Herceptin^®^ to HER-2 receptors

BT-474 cells (1.5 × 10^5^) were washed with ice-cold PBS and harvested. The cells were then incubated with unconjugated native or heated Herceptin^®^ (5 × 10^−6^ to 5 × 10^−2^ mg/mL) in PBS-2% FCS for 30 min at 4°C under gentle agitation. They were then washed twice with PBS-2% FCS, centrifuged (150 g, 5 min, 4°C) and further incubated with FITC-conjugated Herceptin^®^ or isotype control antibody (5 μg/mL) for 30 min at 4°C under gentle agitation. After two additional washing steps, the samples were resuspended in 0.4 mL of PBS-2% FCS. Fluorescence intensity was recorded using the BD FACSCanto II flow cytometer and analyzed using the BD FACS Diva software. A minimum of 10,000 events were analyzed to generate each fluorescence histogram. The gate was arbitrarily set for the detection of FITC fluorescence. Data were presented as mean ± SD from three independent experiments and are fitted with Variable slope model with a 95% confidence interval (GraphPad Prism 6 Software, version 6.01, La Jolla, USA).

### Assessment of total and phosphorylated HER-2 receptor levels

BT-474 cells were seeded in 6-well culture plates at 4×10^5^ cells per well and grown overnight at 37°C in a humidified atmosphere using a 5% CO_2_ incubator. The medium was then removed and cells were washed twice with ice-cold PBS and incubated with or without Herceptin^®^ (50 μg/mL of native or heated antibody). Six days later, the cells were washed twice with ice-cold PBS and lysed with 200 μL RIPA buffer (R0278; Sigma-Aldrich^®^) supplemented with Protease Inhibitor Cocktail (P8340; Sigma-Aldrich^®^) and Phosphatase Inhibitor Cocktail 2 (P5726; Sigma-Aldrich^®^) for 10 min at 4°C. Subsequently, protein lysates were collected with a cell scraper and centrifuged at 12,000 g for 15 min. For each sample, the supernatant containing the cell proteins was recovered and protein concentration was determined using the BCA Protein Assay according to manufacturer’s instructions (23225; Thermo Fisher Scientific, Etten-Leur, The Netherlands).

Protein samples (10 μg/well) were separated by 4–12% Bis-Tris gel (NuPAGE^®^ Novex^®^; Life Technologies™) electrophoresis and transferred onto nitrocellulose membranes (IB3010-02; Life Technologies™) by electroblotting (iBlot^®^ Dry Blotting System; Life Technologies™). Membranes were incubated with 5% bovine serum albumin (BSA, Sigma-Aldrich^®^) in Tris Buffered Saline-Tween solution (TBST; 20 mM Tris, 150 mM NaCl, 0.1% Tween 20, pH 7.6) for 1 h at room temperature (*i.e.,* to block nonspecific binding) under gentle rolling agitation. Blots were washed once with TBST for 15 min and twice for 5 min under gentle rolling agitation. They were then incubated with either anti-HER-2 (1:10000 dilution) or anti-pHER-2 (1:25000 dilution) or anti-ACTIN (*i.e.,* to control equal protein loading; 1:10000 dilution) antibodies in 1% BSA-TBST overnight at 4°C under gentle rolling agitation. The blots were washed thrice for 5 min under gentle rolling agitation. They were then incubated with HRP-conjugated secondary antibody (1:5000 dilution) for 1 h at room temperature under gentle rolling agitation. After additional washing steps, the membranes were developed using Lumi-Light Western Blotting Substrate (Roche Diagnostic Nederland, B.V. Almere, Netherlands) according to the manufacturer’s instructions and visualized using the ChemiDoc™ XRS detection system (Bio-Rad Laboratories B.V., Veenendaal, The Netherlands). Relative densities of HER-2 receptor, phosphorylated HER-2 receptor and ACTIN were measured using densitometric analysis of western-blots with the “Gels” function of ImageJ software. HER-2 receptor and phosphorylated HER-2 receptor were normalized to ACTIN and the fold change in HER-2 receptor and phosphorylated HER-2 receptor over the control condition was determined. Data were presented as mean ± SD from three independent experiments.

### Cell proliferation

BT-474 cells were seeded in 96-well culture plates at 1.5×10^4^ cells per well. The following day, cells were incubated with control and heated Herceptin^®^ (1 × 10^−8^ to 5 × 10^−2^ mg/mL) for 6 days at 37°C in a humidified atmosphere using a 5% CO_2_ incubator. CellTiter 96^®^ non-radioactive cell proliferation assay was carried out according to the manufacturer’s protocol (Promega Benelux B.V., Leiden, the Netherlands). Mean relative growth was expressed as a ratio of the control mean relative growth (vehicle-treated cells). Data were presented as mean ± SD from four independent experiments and are fitted with Variable slope model with a 95% confidence interval (GraphPad Prism 6 Software).

### Statistical analysis

Descriptive statistics was performed using StatPlus^®^:mac (version 5.8.3.8. 2001–2009 Analyst Soft Inc., Vancouver, Canada). Statistical analysis was performed using the non-parametric Mann-Whitney test. Significance was defined as *p* < 0.05 (NS, non-significant; *p < 0.05; **p < 0.01; ***p < 0.001).

## Results

### mHT influence on Herceptin^®^ stability

**FIGURE 1.**
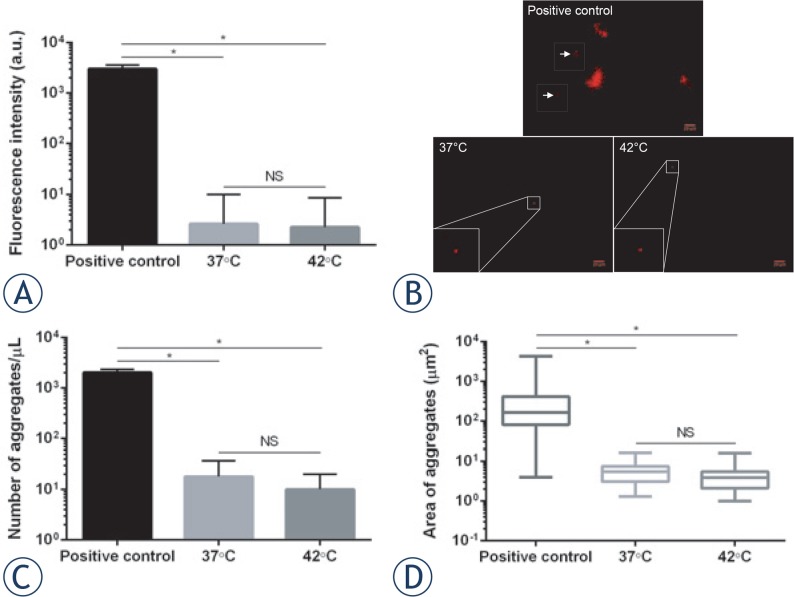
Herceptin^®^ stability. Immediately after Nile Red addition (100 μM), 0.8 mg/mL antibody solution was incubated at 37°C (native antibody), 42°C (heated antibody) and 90°C (positive control) for 1 h. Spectrofluorimetry was used for measuring total fluorescence signal **(A)**, whereas fluorescence images were taken for observing Herceptin^®^ aggregates **(B)**. Representative images of antibody aggregates for each experimental condition are shown. Concentration **(C)** and area **(D)** of antibody aggregates are reported. Data expressed as mean ± SD was calculated from five independent experiments. Statistical analysis was performed using the non-parametric Mann-Whitney test. Significance was defined as *p* < 0.05 (NS, non-significant; *p < 0.05 compared to the positive condition).

The influence of mHT on the Herceptin^®^ stability was investigated by spectrofluorimetry and fluorescence microscopy after Nile Red staining. As shown in Figure 1A, the exposure of Herceptin^®^ to heating at both 37°C and 42°C induced comparable fluorescence levels of Nile Red (3 ± 7 a.u. *vs* 2 ± 7 a.u.; *p* > 0.05), thus suggesting that similar number and/or size of antibody aggregates were formed. To validate this hypothesis, Herceptin^®^ aggregates were observed using fluorescence microscopy (Figure 1B). The incubation of Herceptin^®^ solution at 37°C for 1 h led to fewer (18 ± 10 aggregates/μL) (Figure 1C) and smaller (6 ± 4 μm^2^) (Figure 1D) antibody aggregates than the positive control (2028 ± 156 aggregates/μL, *p* < 0.05; 419 ± 59 μm^2^, *p* < 0.05). mHT did not significantly change the number (10 ± 5 aggregates/μL; *p* > 0.05) and the area (4 ± 4 μm^2^; *p* > 0.05) of Herceptin^®^ aggregates compared to incubation at physiological temperature (Figures 1C–D). These data suggest that mHT does not induce Herceptin^®^ aggregation.

### Immunological properties of heated Herceptin^®^

**FIGURE 2.**
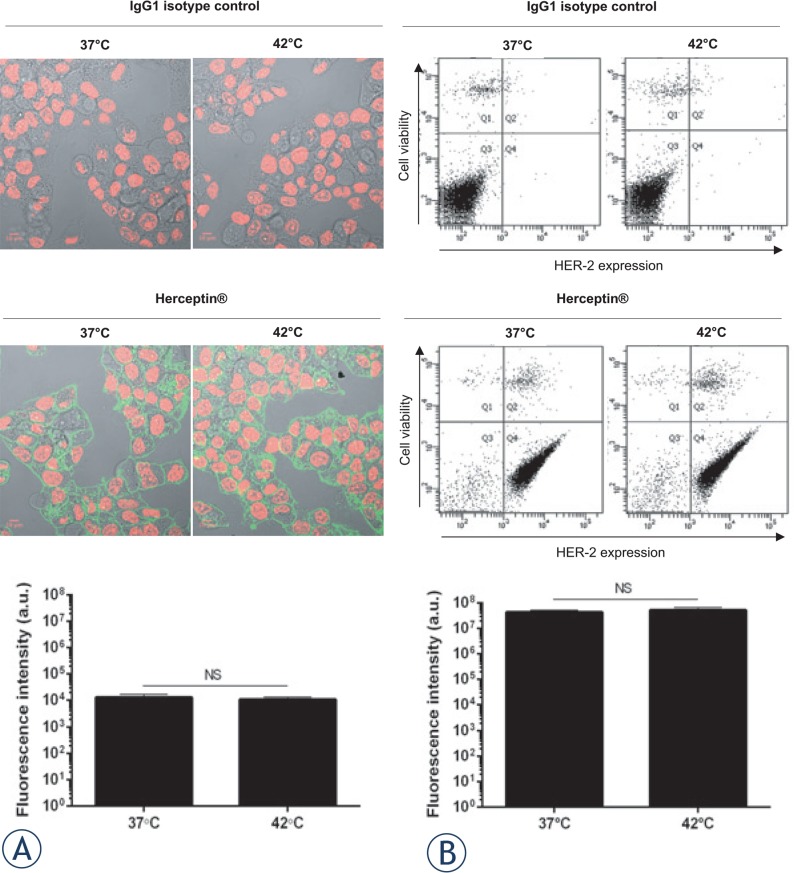
Recognition of HER-2 by Herceptin^®^ following mHT. BT-474 cells were incubated with 10 μg/mL native or heated Herceptin^®^, or matched human isotype control IgG1. Subsequently, the cells were incubated with FITC-conjugated anti-human IgG1-Fc antibody and then analyzed by confocal microscopy **(A)** and flow cytometry **(B)**. Representative images of BT-474 cells and dot-plots of each experimental condition are shown. Data expressed as mean ± SD was calculated from three independent experiments. Statistical analysis was performed using the non-parametric Mann-Whitney test. Significance was defined as *p* < 0.05 (NS, non-significant).

Breast ductal cancer cells, BT-474, which express high levels of HER-2 receptors and are sensitive to Herceptin^®^ have been commonly used for *in-vitro* and *in-vivo* evaluation of Herceptin^®^.^37^,^38^ Therefore, to determine whether mHT altered the recognition of HER-2 receptors by Herceptin^®^, confocal fluorescence microscopy and flow cytometry analyses were performed on BT-474 cells. Confocal fluorescence images of BT-474 cells revealed a similar percentage of HER-2 positive cells (98 ± 2% *vs* 99 ± 1%; *p* > 0.05) and fluorescence levels (1.3×10^4^ ± 1.3×10^3^ a.u. *vs* 1.2×10^4^ ± 0.6×10^3^ a.u.; *p* > 0.05), whether these cells were stained with native (*i.e.,* 37°C, 1 h) or heated (*i.e.,* 42°C, 1 h) Herceptin^®^ (Figure 2A). No HER-2 positive cells were detected with human IgG1 control isotype antibody. In addition, flow cytometry analyses supported these results (Figure 2B). Indeed, the dot plots of BT-474 cells were unchanged with comparable percentages of living HER-2 positive cells (84 ± 3% *vs* 88 ± 3%; *p* > 0.05) and fluorescence intensities (5.8×10^3^ ± 6×10^2^ a.u. *vs* 6.1×10^3^ ± 4×10^2^ a.u.; *p* > 0.05) after labeling with native and heated Herceptin^®^ (Figure 2B).

**FIGURE 3.**
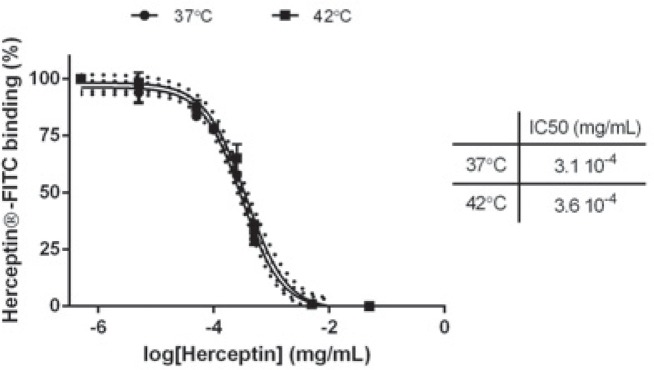
Binding affinity of Herceptin^®^ to HER-2. BT-474 cells were first incubated with unconjugated Herceptin^®^ (5 × 10^−6^ to 5 × 10^−2^ mg/mL) and subsequently with FITC-Herceptin^®^. Fluorescence intensity on flow cytometry is plotted as a function of unlabeled Herceptin^®^ concentration used for receptor saturation. Data expressed as mean ± SD calculated from three independent experiments and are fitted with Variable slope model (solid curve; confidence intervals, dotted curve) with a 95% confidence interval. Statistical analysis was performed using the non-parametric Mann-Whitney test. Significance was defined as *p* < 0.05 (NS, non-significant).

To gain insight into the effect of mHT on the immunological properties of Herceptin^®^, the binding affinity of this antibody to HER-2 receptors was assessed by a competition assay on BT-474 cells using FITC-coupled Herceptin^®^. Figure 3 indicates that low concentrations of unlabeled and native Herceptin^®^ (*i.e.,* below 5×10^−5^ mg/mL) did not change the binding of FITC-conjugated Herceptin^®^ to HER-2 receptors, while a concentration range from 1×10^−4^ mg/mL to 5×10^−2^ mg/mL of this antibody induced a significant inhibition of the binding of FITC-coupled Herceptin^®^. Heated Herceptin^®^ led to a similar inhibition of FITC-conjugated Herceptin^®^ as native Herceptin^®^. Moreover, native and heated Herceptin^®^ show comparable IC50 values, *i.e.,* 3.1×10^−4^ mg/mL and 3.6×10^−4^ mg/mL (*p* > 0.05), respectively. These results indicate that mHT does not modify the recognition and the binding affinity of Herceptin^®^ to HER-2 receptors.

### Pharmacological properties of heated Herceptin^®^

**FIGURE 4.**
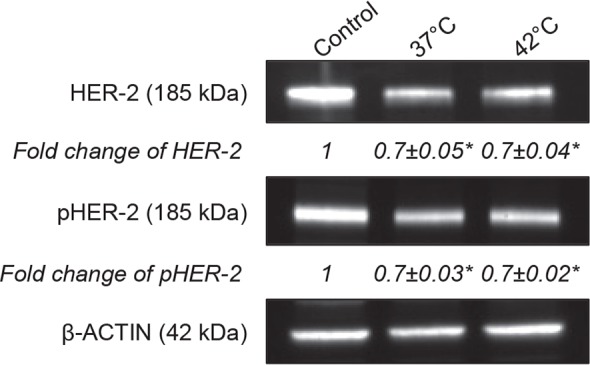
Degradation and Dephosphorylation of HER-2 receptors by Herceptin^®^. 4×10^5^ BT-474 cells were seeded in a 6-well plate and treated with Herceptin^®^ (50 μg/mL) for 6 days. Immunoblots of protein lysates were analyzed for total and phosphorylated HER-2 receptors. Representative immunoblots of one experiment out of three independent experiments are shown. The number at the bottom of each lane indicates the relative fold change versus the control after normalization with the β-ACTIN signal. Data expressed as mean ± SD calculated from three independent experiments. Statistical analysis was performed using the non-parametric Mann-Whitney test. Significance was defined as *p* < 0.05 (NS, non-significant; *p < 0.05 compared to the positive condition).

To investigate how the binding of heated Herceptin^®^ to HER-2 receptors affects them, their total and phosphorylated levels were monitored using western-blot assay. As expected, the exposure of BT-474 cells to native Herceptin^®^ (50 μg/mL) resulted in significant decrease in relative densities of HER-2 receptors and phosphorylated HER-2 receptors compared to untreated cells (*p* < 0.05) (Figure 4). These results suggest that native Herceptin^®^ leads to the internalization and the degradation of HER-2 receptors, thus preventing their phosphorylation in BT-474 cells. In addition, heated Herceptin^®^ led to a decrease in total and phosphorylated HER-2 receptor levels that were comparable to the native Herceptin^®^ (*p* > 0.05) (Figure 4).

**FIGURE 5.**
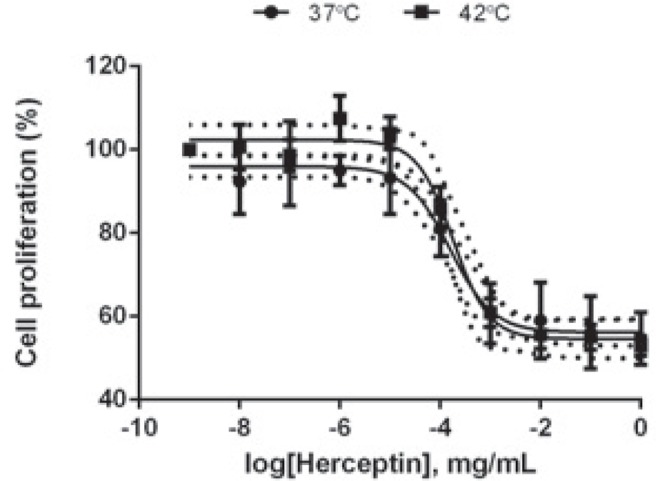
Effect of Herceptin^®^ on BT-474 cell proliferation. BT-474 cells were seeded in a 96-well plate at 15,000 cells per well and were treated with native or heated Herceptin^®^ (1×10^−8^ - 5×10^−2^ mg/mL) for 6 days. Cell proliferation assay was performed. The mean relative growth was expressed as a ratio of the mean control relative growth (vehicle-treated cells). Data expressed as mean ± SD calculated from four independent experiments and are fitted with Variable slope model (solid curve; confidence intervals, dotted curve) with a 95% confidence interval. Statistical analysis was performed using the non-parametric Mann-Whitney test. Significance was defined as *p* < 0.05 (NS, non-significant).

Using a cell proliferation assay, the anti-proliferative effects of native and heated Herceptin^®^ in BT-474 cells were investigated 6 days after antibody treatment. As shown in Figure 5, low native Herceptin^®^ concentration (1×10^−8^ mg/mL – 1×10^−5^ mg/mL) slightly affected BT-474 cell proliferation, while antibody concentrations above 1×10^−4^ mg/mL induced a significant decrease in cell proliferation. Heated Herceptin^®^ was similarly potent in suppressing BT-474 cell proliferation (Figure 5). These results suggest that mHT has no significant effect on the anti-proliferative properties of Herceptin^®^.

## Discussion

The present study investigated whether mHT (42°C for 1 h) influences the stability, the immunological and pharmacological properties of Herceptin^®^ *invitro*. First, we showed that mHT did not result in additional Herceptin^®^ aggregates compared to the physiological temperature (Figure 1). In agreement with published data, the low number of aggregates observed reflects the good Herceptin^®^ stability under our mHT exposure conditions.^33^,^34^ Indeed, based on differential scanning calorimetry measurements, previous studies showed that only temperatures above 50°C induced significant number of antibody aggregates.^39^ However, temperatures between 43°C and 50°C have been shown to cause edema, vascular occlusion and hemorrhage.^40^,^41^ Thus, the use of appropriate mHT is of great importance with respect to Herceptin^®^ stability and tissue physiology, since the formation of antibody aggregates and tissue damage may induce loss of therapeutic activity^42^,^43^ and severe side effects^39^,^40^, respectively. In addition, the antibody aggregation alters the immunogenicity of the therapeutic antibody. As a result, immune tolerance existing natively to self-antigens (*e.g.,* therapeutic antibodies) breaks down and aggregate-targeting antibodies are produced.^43^ These antibodies can either have no significant consequences for the patients, or decrease the therapeutic effectiveness of the therapeutic antibodies.

Previous investigations reported that mHT has few incidences on the immunological properties.^44^,^45^ Indeed, Hauck *et al.* reported that no obvious effects of temperature on kinetic binding parameters of the anti-Tenascin antibody (*i.e.,* 81C6) to human glioma cells were apparent between 37°C and 45°C.^44^ In agreement with these studies, we found that the exposure of Herceptin^®^ to mHT did not modify the recognition and the binding affinity of Herceptin^®^ to HER-2 receptors using human HER-2 positive breast cancer cells such as BT-474 (Figures 2, 3), indicating that mHT has no significant effects on the immunological properties of this therapeutic antibody. The Herceptin^®^ binding to HER-2 receptors is expected to induce their internalization^7^,^8^, leading to their subsequent degradation.^38^,^46^ In agreement with these works^46^, we showed that the exposure of BT-474 cells to native Herceptin^®^ as well as heated Herceptin^®^ led to a similar decrease in total and phosphorylated HER-2 receptor levels (Figure 4). As previously shown^35^,^38^, this Herceptin^®^-mediated interference with the HER-2 transduction pathway results in a potent inhibition of the growth of HER-2 positive breast cancer cells (Figure 5). Finally, our data revealed that the anti-proliferating properties of Herceptin^®^ were maintained after mHT.

Since it is preserving the stability, and the immunological and pharmacological properties of Herceptin^®^, mHT could represent a promising strategy for local delivery of Herceptin^®^ in patients with HER-2 positive breast cancer. Indeed, this method is fully compatible with i.v. administration of Herceptin^®^. In this scenario, the breast tumor would be exposed to local mHT using high intensity focused ultrasound, radiofrequency or infrared radiation.^47^ As the tumor perfusion and the vascular permeability increase as a result of applying mHT and the interstitial fluid pressure is reduced, an improved i.t. Herceptin^®^ bioavailability could be obtained. As a result, the tumor response to Herceptin^®^-based immunotherapy might be enhanced using this method. This hypothesis is supported by preclinical studies, which reported that combining mHT with intact radiolabelled mAbs improved the tumor mAb uptake^48^, as well as an increase in radio-immunotherapy efficacy in rodent xenograft models.^31^,^48^ Heating methods, especially focused ultrasound, will be investigated and compared to water-bath in future studies.

## Conclusions

In summary, our findings suggest that mHT does not lead to significant changes in the recognition to and the binding affinity of Herceptin^®^ to HER-2 receptors. In addition, the ability of Herceptin^®^ to inhibit the HER-2 signaling pathway and the anti-proliferative properties of Herceptin^®^ remain unchanged. These results are in line with the observed stability of Herceptin^®^ with respect to exposure to mHT. Thus, mHT can also safely be used to improve the i.t. bioavailability of Herceptin^®^ as is already done for nanoparticles.^49^ Further *in-vivo* investigations are required to evaluate the influence of mHT on the anti-tumor effectiveness and the side effects of Herceptin^®^ after exposure of tumors to mHT.
